# L-Citrulline Supplementation Improves Arterial Blood Flow and Muscle Oxygenation during Handgrip Exercise in Hypertensive Postmenopausal Women

**DOI:** 10.3390/nu16121935

**Published:** 2024-06-19

**Authors:** Yejin Kang, Katherine N. Dillon, Mauricio A. Martinez, Arun Maharaj, Stephen M. Fischer, Arturo Figueroa

**Affiliations:** 1 Department of Kinesiology and Sport Management, Texas Tech University, Lubbock, TX 79409, USA; yejin.kang@ttu.edu (Y.K.); katherine.dillon@ttu.edu (K.N.D.); mauricio.martinez@ttu.edu (M.A.M.); 2 Department of Epidemiology and Cancer Control, St. Jude Children’s Research Hospital, Memphis, TN 38105, USA; arun.maharaj@stjude.org; 3 Department of Family and Community Medicine, The University of Texas Health Science Center at Houston, Houston, TX 77030, USA; stephen.fischer@uth.tmc.edu

**Keywords:** L-citrulline, endothelial function, muscle oxygenation, blood flow, vascular conductance, handgrip exercise, postmenopausal women

## Abstract

Endothelial dysfunction decreases exercise limb blood flow (BF) and muscle oxygenation. Acute L-Citrulline supplementation (CIT) improves muscle tissue oxygen saturation index (TSI) and deoxygenated hemoglobin (HHb) during exercise. Although CIT improves endothelial function (flow-mediated dilation [FMD]) in hypertensive women, the impact of CIT on exercise BF and muscle oxygenation (TSI) and extraction (HHb) are unknown. We examined the effects of CIT (10 g/day) and a placebo for 4 weeks on blood pressure (BP), arterial vasodilation (FMD, BF, and vascular conductance [VC]), and forearm muscle oxygenation (TSI and HHb) at rest and during exercise in 22 hypertensive postmenopausal women. Compared to the placebo, CIT significantly (*p* < 0.05) increased FMD (Δ−0.7 ± 0.6% vs. Δ1.6 ± 0.7%) and reduced aortic systolic BP (Δ3 ± 5 vs. Δ−4 ± 6 mmHg) at rest and improved exercise BF (Δ17 ± 12 vs. Δ48 ± 16 mL/min), VC (Δ−21 ± 9 vs. Δ41 ± 14 mL/mmHg/min), TSI (Δ−0.84 ± 0.58% vs. Δ1.61 ± 0.46%), and HHb (Δ1.03 ± 0.69 vs. Δ−2.76 ± 0.77 μM). Exercise BF and VC were positively correlated with improved FMD and TSI during exercise (all *p* < 0.05). CIT improved exercise artery vasodilation and muscle oxygenation via increased endothelial function in hypertensive postmenopausal women.

## 1. Introduction

Endothelial function is characterized by adequate nitric oxide (NO) bioavailability [1], an essential vasodilator for normal vascular tone and blood flow (BF) regulation [2]. Endothelial cells are stimulated via an increase in BF induced by shear stress [3], leading to the catabolism of L-arginine (ARG) into NO by endothelial NO synthase (eNOS) [4]. NO increases BF in conduit arteries (macrovascular) [5] and resistance arterioles (microvascular) at rest [6] via the relaxation of vascular smooth muscle cells [2]. Conduit arteries are responsible for maintaining a steady BF to resistance arterioles, while arterioles regulate oxygen and nutrient delivery to capillaries [7]. Menopause and hypertension are associated with macro- and microvascular endothelial dysfunction at rest due to ARG and NO deficiency, leading to reduced vasodilatory capacity and arterial stiffness [8,9,10,11]. Microvascular dysfunction in hypertension leads to impaired muscle BF and end organ damage, contributing to increased cardiovascular event risk [11].

During exercise, active skeletal muscles require sufficient oxygen delivery to match metabolic demands; thus, increasing BF to the contracting muscles is essential [12,13]. Muscle BF during exercise is regulated by the balance between local vasodilators, including NO and muscle metabolites, and vasoconstriction via sympathetic activity [14]. Aging and hypertension lower NO availability and impair the ability of contracting muscles to blunt sympathetic vasoconstriction during exercise (functional sympatholysis), reducing BF and oxygen delivery to skeletal muscles [15]. The tissue oxygen saturation index (TSI), an indicator of perfusion and oxygen delivery to skeletal muscles [16,17], decreases during exercise in older women, indicating microvascular dysfunction and impaired vasodilatory capacity [17]. NIRS-derived deoxygenated hemoglobin (HHb) content reflects local muscle oxygen extraction as the ratio of oxygen utilization to the oxygen supply within the microvasculature [18,19]. Diminished BF in older adults [19] and NOS inhibition [20] cause increased muscle oxygen extraction (measured as HHb) during exercise to meet the oxygen demand for mitochondrial oxidative energy production. Taken together, impaired NO may contribute to reductions in arterial BF and muscle oxygenation during exercise [11,15] in hypertensive postmenopausal women.

NO, a potent vasodilator, can be synthesized through the conversion of ARG via eNOS [21], which plays a major role in regulating muscle BF and oxygen delivery during exercise [22]. While ARG supplementation can increase plasma ARG concentration and macrovascular endothelial function at rest [23], it is not optimal to produce NO due to stimulation of arginase activity [24], leading to no effects on oxygen delivery and consumption during exercise [25]. Unlike oral ARG, the majority of ingested L-citrulline (CIT) bypasses arginase catabolism in the intestines and the liver, and is converted to ARG in the kidneys [26]. Thus, CIT supplementation is more efficient to enhance ARG and NO availability than oral ARG supplementation [27]. Comparing increasing doses of CIT (2–15 g), a 10 g dose was well tolerated and resulted in greater plasma ARG availability in the elderly [28], suggesting this could be the most appropriate dose for clinical use [29]. Improvements in macro- and microvascular endothelial function at rest following CIT supplementation were observed in hypertensive postmenopausal women [30] and patients with heart failure with preserved ejection fraction [31] by enhancing ARG availability. CIT supplementation for 7 days showed higher TSI and lower HHb in the leg muscle during cycling exercise in young active men, suggesting improved microvascular perfusion and reduced oxygen consumption [25]. Although CIT may enhance arterial vasodilatory capacity at rest and muscle oxygenation responses to exercise in young men, the efficacy of CIT supplementation to improve macrovascular function and muscle oxygenation during exercise in postmenopausal women with hypertension is unknown. Thus, the purpose of this study was to examine the impact of 4 weeks of CIT supplementation on brachial artery vasodilatory capacity and muscle oxygenation at rest and during handgrip exercise in hypertensive postmenopausal women. We hypothesized that 4 weeks of CIT would improve arterial vasodilation, muscle oxygenation, and muscle extraction during exercise via improved brachial artery endothelial-dependent vasodilation.

## 2. Materials and Methods

### 2.1. Participants

Postmenopausal women (at least 1 year without menstruation), aged 50 to 71 years, were enrolled in this study. All participants were hypertensive (resting systolic blood pressure (SBP) ≥ 130 mmHg or ≥120 mmHg if they were on anti-hypertensive medication) and sedentary (<120 min/week of exercise). Hypertension was defined according to the 2017 American Heart Association guidelines [32], which differ from international guidelines (SBP > 140 mmHg). Exclusion criteria included a body mass index > 40 kg/m^2^, SBP ≥ 160 mmHg, current use of tobacco, or >7 alcoholic drinks consumed per week. Participants were excluded if they were diagnosed with cardiovascular diseases, type 1 or 2 diabetes, or any metabolic/chronic diseases or if they were taking beta-blockers, more than one vasoactive drug, or dietary supplements with vasodilatory and/or antioxidant effects. No participants changed their medications at least 3 months prior to the study participation and during the study period. Participants signed a written informed consent and completed a health questionnaire. All study protocols were explained by a researcher and participants were familiarized with the protocols prior to the experimental visits. All procedures were approved by the Texas Tech University Institutional Review Board (IRB2018-463; approved on 31 May 2019) and registered in ClinicalTrials.gov https://clinicaltrials.gov/study/NCT05227781 (accessed on 7 February 2022). under NCT05227781.

### 2.2. Experimental Protocol

This study was of a double-blind, randomized, placebo-controlled, and parallel design. Measurements were performed in the morning after an overnight fast of at least 8 h. Participants refrained from caffeine and prescription medications (~12 h) before each visit, and from alcohol and physical activity for at least 24 h. Following at least 20 min of rest in the supine position, vascular measurements were performed in a quiet, temperature-controlled, dimly lit (~23 °C) room. All measurements were collected 4 weeks after the supplementations at the same time of the day (±1 h) following the sequence utilized at baseline.

The principal investigator, who was not involved in data collection, performed the group randomization stratified by age and SBP using an online program. Participants were randomized to consume either CIT (10 g/day) (*n* = 11) or the placebo (maltodextrin) (*n* = 11) for 4 weeks (NOW^®^ Foods). Participants consumed 4.5 g (6 capsules of 750 mg) and 5. 25 g (7 capsules of 750 mg) in the morning and evening, respectively, for 4 weeks. Participants were asked to not consume foods containing high levels of ARG and CIT (e.g., nuts, almonds, watermelon) or supplements containing antioxidants during the study duration. Adherence to the supplements was calculated by counting capsules from the returned bottles. Participants were asked to keep their habitual diet and physical activity until the completion of the study.

### 2.3. Measurements

#### 2.3.1. Anthropometrics

Height (m) was measured using a stadiometer (Free-Standing Portable Height Rod, Detecto, Webb City, MO, USA) to the nearest 0.01 m. Weight (kg) was obtained using a beam scale (Weigh Beam, Detecto, Webb City, MO, USA) to the nearest 0.1 kg. The body mass index was calculated by dividing weight (kg) by height squared (m^2^). Waist circumference was measured with a non-elastic tape measure at the point between the last rib and the upper border of the iliac crest [33].

#### 2.3.2. Forearm Muscle Strength and Dynamic Handgrip Exercise

Muscle strength of the dominant hand was determined through maximal voluntary contraction (MVC) using a digital handgrip dynamometer (Lafayette Instrument Co., Lafayette, IN, USA). Participants performed three MVCs with a minute break between trials, and the highest value was considered as the MVC. Following the collection of resting measurements, participants performed a rhythmic handgrip exercise at 30% of MVC for 3 min with a metronome-controlled rate (3-s concentric/3-s eccentric). Participants had continuous visual guidance of the target force on the screen and verbal feedback from the researchers to keep on the target MVC during exercise.

#### 2.3.3. Brachial and Aortic Blood Pressure and Arterial Stiffness

Following 20 min of rest in the supine position, brachial BP was measured at least twice using an automated oscillometric device (HEM-907XL; Omron Healthcare, Vernon Hill, IL, USA) and an average of two measures with a difference of less than 5 mmHg was used in the analysis. An arterial tonometer (SphygmoCor CPV, AtCor Medical, Sydney, Australia) was placed on the radial artery to collect pressure waveforms. Radial waveforms were calibrated with brachial diastolic BP (DBP) and mean arterial pressure (MAP) to generate aortic pressure waveforms. Brachial and aortic SBP, DBP, MAP, and pulse pressure (PP) were determined at rest. Brachial BP was measured once at minute 3 of handgrip exercise. Changes (Δ) in SBP, DBP, MAP, and PP were calculated from rest to minute 3 of exercise. Carotid–femoral pulse wave velocity (cfPWV), a measure of aortic stiffness, was assessed using wave sensors (Complior Analyse, Alam Medical, Vincennes, France) positioned over the common carotid and femoral arteries. The distance between the two arterial points was measured above the body surface using a non-elastic segmometer. The value of cfPWV was determined by dividing the distance of the carotid-femoral segment by the transit time between the two pulse waves. At least two cfPWV readings were obtained and averaged if there was a ≤0.3 m/s difference between two values.

#### 2.3.4. Brachial Artery Flow-Mediated Dilation (FMD) and Hemodynamics

A 12 MHz linear array Doppler ultrasound probe (Logiq S7, General Electric, Milwaukee, WI, USA) was placed on the brachial artery (2–3 cm proximal to the antecubital fossa) and held with a probe holder at an insonation angle < 60°. The baseline diameter was recorded for 2 min, and the cuff was inflated to 250 mmHg for 5 min using a rapid-inflating cuff (Hokanson E20, Bellevue, WA, USA) placed on the right forearm distal to the brachial artery. Arterial occlusion was followed by rapid cuff deflation and 3 min of reactive hyperemia. The brachial artery diameter and mean blood velocity were continuously recorded during the 10 min protocol. Images were recorded through an online video software (OBS Studio version 29.1.3). The images were analyzed using automated edge detection software (Quipu Cardiovascular Suite version 3.6.0, Pisa, Italy), and brachial artery flow-mediated dilation (FMD) was calculated by using the following formula.

FMD (%) = (peak diameter − baseline diameter)/baseline diameter × 100

During handgrip exercise protocol, the ultrasound probe was placed on the upper arm, and the brachial artery diameter and mean blood velocity were continuously recorded at rest and during 3 min of exercise. Forearm muscle BF (FBF) and VC (FVC) were calculated as described in the previous study [34] and averaged by every 1 min for data analysis. Changes (Δ) in FBF and FVC were calculated from rest (0 min) to the 1st, 2nd, and 3rd minute of exercise. ΔFBF and ΔFVC were averaged over the 3 min (Δ3-min) of exercise.

FBF (mL/min) = mean blood velocity (cm/sec) × π (brachial artery diameter (cm)/2)2 × 60

FVC (mL/min/mmHg) = (FBF (mL/min)/MAP (mmHg)) × 100

#### 2.3.5. Muscle Oxygenation

Forearm muscle oxygenation was measured using a frequency domain, non-invasive, near-infrared spectroscopy (NIRS) system (PortaMon, Artinis Medical System BV, Elst, Gelderland, The Netherlands) positioned on the skin over the flexor digitorum profundus of the right hand. The optodes were covered and stabilized by wrapping with a black elastic bandage around the forearm. The NIRS-derived data were acquired by transmitting to a computer via Bluetooth at 10 Hz, and recorded using software (Oxysoft version 3.0, Artinis Medical Systems BV, Elst, The Netherlands). The NIRS device continuously monitored the relative changes in oxygenated hemoglobin (O_2_Hb) and HHb from rest to exercise. The HHb reflects the balance between the local oxygen supply and utilization and provides an estimate of changes in fractional oxygen extraction [18]. TSI is an absolute measure of muscle oxygenation [35] and was calculated as follows: TSI (%) = (O_2_Hb/(O_2_Hb + HHb)) × 100. Changes (Δ) in TSI, HHb, and O_2_Hb were calculated from rest to minutes 1, 2, and 3 of exercise. ΔTSI, ΔHHb, and ΔO_2_Hb were averaged over the 3 min (Δ3-min) of exercise.

### 2.4. Statistical Analysis

Based on previous studies that showed increased FBF [36] and higher O_2_Hb concentration [37] during exercise after the acute ingestion of an NO precursor in young adults, 10 participants per group were estimated to detect a significant difference in muscle oxygenation with ≥80% power at the α = 0.05 level. All statistical analyses were performed with SPSS 29.0 (IBM SPSS Statistics, Chicago, IL, USA). The normality of the data was tested using the Shapiro–Wilk test. Between-group differences at 0 week were compared using an independent *t*-test. Two-way repeated measures analysis of variance (ANOVA) with Bonferroni adjustments were used to detect significant changes in brachial and aortic BP, FMD, and cfPWV between groups (placebo and CIT) over time (0 week and 4 weeks). Two-way repeated measures ANOVA with Bonferroni adjustments were used to determine significant differences in arterial vasodilation (ΔFBF and ΔFVC) and NIRS-derived (ΔTSI, ΔHHb, and ΔO_2_Hb) responses to exercise (0, 1, 2, 3 min) from 0 to 4 weeks between groups. When a significant group-by-time interaction was detected, pairwise comparisons were performed through the Bonferroni adjustment that corrects the error of multiple comparisons. An independent *t*-test was performed to determine significant differences of average changes over the 3 min of exercise on arterial vasodilation (Δ3-min_FBF and Δ3-min_FVC) and NIRS-derived measures (Δ3-min_TSI, Δ3-min_HHb, and Δ3-min_O_2_Hb) from 0 to 4 weeks between groups. Pearson’s correlation was performed to identify relationships between the changes in FMD, arterial vasodilation (Δ3-min_FBF and Δ3-min_FVC), and muscle oxygenation measures (Δ3-min_TSI, Δ3-min_HHb, and Δ3-min_O_2_Hb) during exercise from 0 to 4 week. Data were presented as mean ± standard deviation (SD) in tables and standard error (SE) in figures. Statistical significance was set at *p* < 0.05.

## 3. Results

**Figure 1:**
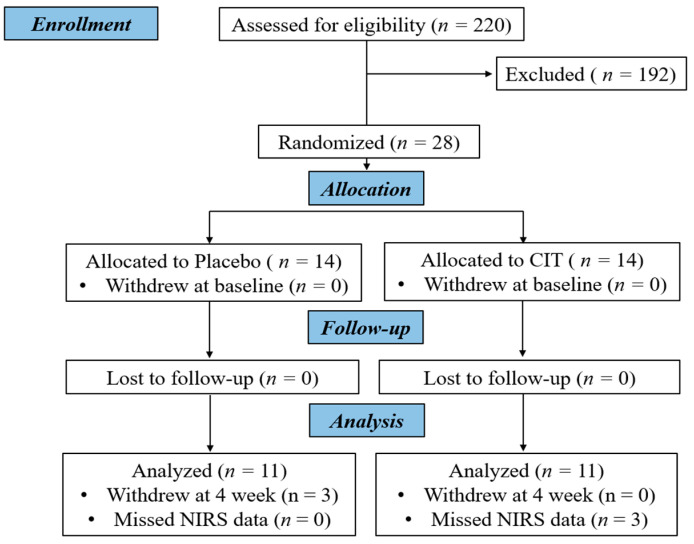
CONSORT flow chart of participants through the study. CIT, L-citrulline; NIRS, near-infrared spectroscopy.

Twenty-two participants finished the study (Figure 1). Compliance with the supplements were 94.2 ± 2.0% (placebo) and 93.7 ± 2.2% (CIT). No adverse effects were reported during the study. Participant characteristics and medications are shown in Table 1. There were no significant baseline differences between the groups (all *p* > 0.05, Table 1). Participants in both groups were on antihypertensive medications (PL = 3 and CIT = 4) and statins (PL = 1 and CIT = 1).

### 3.1. Effects of Supplementations on Blood Pressure, Endothelial Function, and Arterial Stiffness

**Figure 2:**
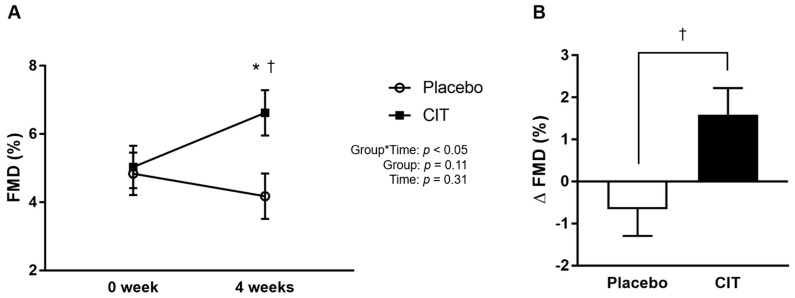
Brachial artery flow-mediated dilation (FMD) at 0 and 4 weeks (**A**) and change (Δ) in FMD from 0 to 4 weeks (**B**). Abbreviations: CIT, L-citrulline. Data are means ± standard error (SE). * *p* < 0.05 vs. 0 week; ^†^ *p* < 0.05 vs. placebo.

There were no group differences in cfPWV, FMD, resting brachial and aortic BP, and brachial BP responses to exercise at 0 week (all *p* > 0.05, Table 2). No significant group-by-time interaction was observed for cfPWV (*p* > 0.05, Table 2). There was a significant group-by-time interaction for brachial FMD (*p* < 0.05, Figure 2A). Compared to the placebo, FMD significantly increased after 4 weeks of CIT supplementation (placebo: Δ−0.7 ± 0.6% vs. CIT: Δ1.6 ± 0.7%, *p* < 0.05) (Figure 2B).

There were significant group-by-time interactions in resting brachial and aortic SBP and MAP (all *p* < 0.05, Table 2). Four weeks of CIT supplementation significantly reduced brachial SBP (placebo: Δ2 ± 5 vs. CIT: Δ−4 ± 6 mmHg), brachial MAP (placebo: Δ2 ± 4 vs. CIT: Δ−2 ± 4 mmHg), aortic SBP (placebo: Δ3 ± 5 vs. CIT: Δ−4 ± 6 mmHg), and aortic MAP (placebo: Δ2 ± 4 vs. CIT: Δ−3 ± 5 mmHg) compared to the placebo (all *p* < 0.05, Table 2). No significant group-by-time interactions were observed for brachial BP responses to exercise (all *p* > 0.05, Table 2).

### 3.2. Effects of Supplementations on Arterial Vasodilation during Exercise

**Figure 3:**
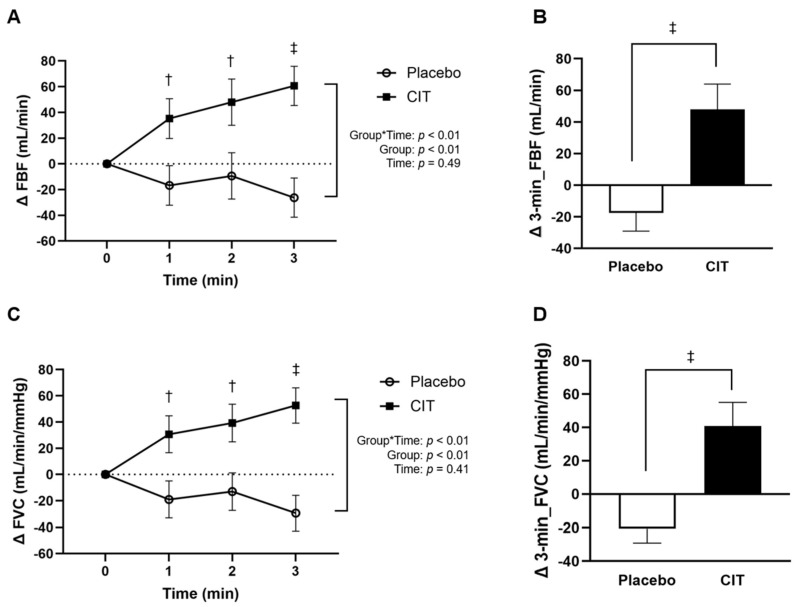
Changes (Δ) in forearm blood flow (FBF) (**A**) and vascular conductance (FVC) (**C**) during 3 min exercise from 0 to 4 weeks, and average changes in FBF (Δ3-min_FBF) (**B**) and FVC (Δ3-min_FVC) (**D**) over the 3 min of exercise from 0 to 4 weeks. Abbreviation: CIT, L-citrulline. Data are means ± standard error (SE). ^†^ *p* < 0.05; ^‡^ *p* < 0.01 vs. placebo.

There were no group differences in the absolute values of FBF and FVC at rest at 0 and 4 weeks (all *p* > 0.05, Table 3). Significant group-by-time interactions were found for ΔFBF and ΔFVC (both *p* < 0.01). ΔFBF and ΔFVC were significantly enhanced during the whole exercise period (all *p* < 0.05, Figure 3A,C). Compared to the placebo, Δ3-min_FBF (placebo: Δ−17 ± 12 vs. CIT: Δ 48 ± 16 mL/min, *p* < 0.01, Figure 3B) and Δ3-min_FVC (placebo: Δ−21 ± 9 vs. CIT: Δ41 ± 14 mL/mmHg/min vs. *p* < 0.01, Figure 3D) during exercise were significantly higher after CIT.

### 3.3. Effects of Supplementations on Muscle Oxygenation Responses to Exercise

**Figure 4:**
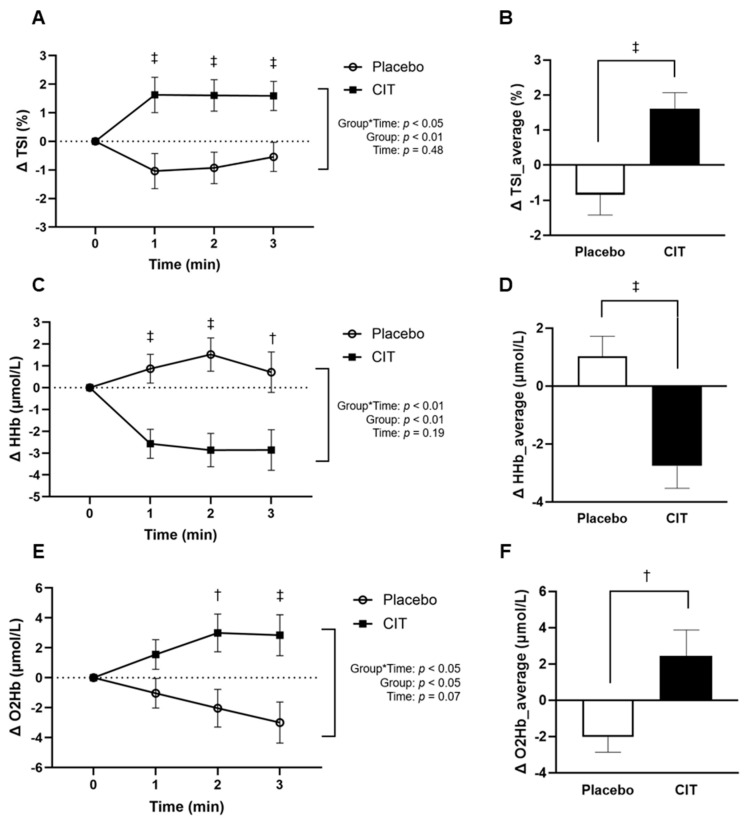
Changes (Δ) in the tissue oxygen saturation index (TSI) (**A**), deoxygenated hemoglobin (HHb) (**C**), and oxygenated hemoglobin (O_2_Hb) (**E**) during handgrip exercise from 0 to 4 weeks, and average changes in TSI (Δ3-min_TSI) (**B**), HHb (Δ3-min_HHb) (**D**), and O_2_Hb (Δ3-min_O_2_Hb) (**F**) over the 3 min of exercise from 0 to 4 weeks. Abbreviation: CIT, L-citrulline. Data are means ± standard error (SE). ^†^ *p* < 0.05 vs. placebo; ^‡^ *p* < 0.01 vs. placebo.

There were no group differences in the absolute values of TSI, HHb, and O_2_Hb at rest at 0 and 4 weeks (all *p* > 0.05, Table 4). There were significant group-by-time interactions for ΔTSI, ΔHHb, and ΔO_2_Hb during exercise (all *p* < 0.05, Figure 4). ΔTSI significantly increased during the whole exercise period after CIT compared to the placebo (all *p* < 0.05, Figure 4A). Compared to the placebo, Δ3-min_TSI during exercise was significantly higher after CIT (placebo: Δ−0.84 ± 0.58 vs. CIT: Δ1.61 ± 0.46%, *p* < 0.01) (Figure 4B). ΔHHb was significantly lower throughout the exercise after CIT compared to the placebo (all *p* < 0.05, Figure 4C). Compared to the placebo, Δ3-min_HHb during exercise was lower after CIT (placebo: Δ1.03 ± 0.69 vs. CIT: Δ −2.76 ± 0.77 μM, *p* < 0.01) (Figure 4D). Despite no significant between-group difference at minute 1, ΔO_2_Hb significantly increased during minutes 2 and 3 of exercise after CIT compared to the placebo (all *p* < 0.05, Figure 4E). Compared to the placebo, Δ3-min_O_2_Hb during exercise was higher after CIT (placebo: Δ −2.02 ± 0.84 vs. CIT: Δ 2.46 ± 1.42 μM, *p* < 0.05) (Figure 4F).

### 3.4. Correlations between FMD with Arterial Vasodilation and Muscle Oxygenation after CIT Supplementation

**Figure 5:**
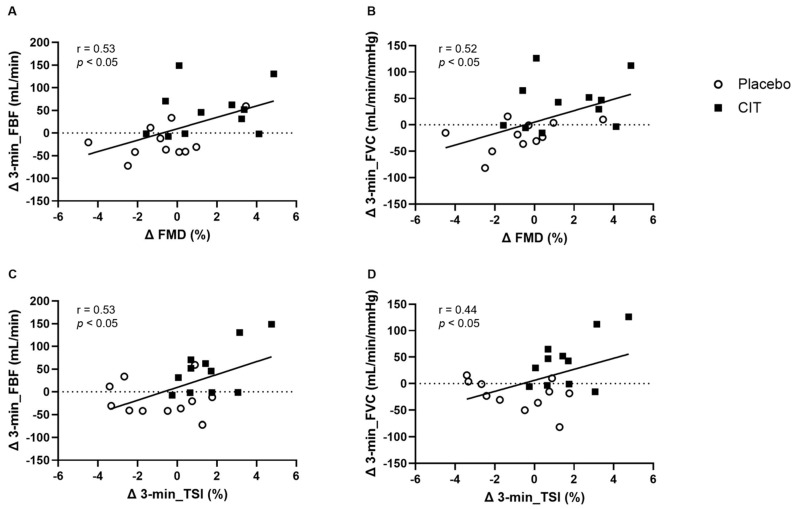
Correlations between average changes over the 3 min of exercise (Δ3-min) in forearm blood flow (Δ3-min_FBF) and vascular conductance (Δ3-min_FVC) from 0 to 4 weeks with changes in brachial artery flow-mediated dilation (ΔFMD) from 0 to 4 weeks (**A**,**B**) and average changes in the tissue oxygen saturation index (Δ3-min_TSI) from 0 to 4 weeks (**C**,**D**).

Enhanced Δ3-min_FBF (r = 0.53, *p* < 0.05, Figure 5A) and Δ3-min_FVC (r = 0.52, *p* < 0.05, Figure 5B) during exercise from 0 to 4 weeks were correlated with ΔFMD from 0 to 4 weeks. Δ3-min_NIRS-derived measures were not significantly correlated with ΔFMD from 0 to 4 weeks. Moreover, improved Δ3-min_FBF was correlated with Δ3-min_TSI (r = 0.53, *p* < 0.05, Figure 5C), Δ3-min_O_2_Hb (r = 0.54, *p* < 0.05), but not with Δ3-min_HHb during exercise (*p* > 0.05). Improved Δ3-min_FVC was correlated with Δ3-min_TSI (r = 0.44, *p* < 0.05, Figure 5D) Δ3-min_O_2_Hb (r = 0.62, *p* < 0.01), and Δ3-min_HHb during exercise (r = −0.44, *p* < 0.05).

## 4. Discussion

Our findings indicate that CIT supplementation increased brachial FMD and reduced brachial and aortic SBP and MAP at rest. The novel findings of this study are that 4 weeks of CIT supplementation increased FBF, FVC, TSI, and O_2_Hb and attenuated HHb during dynamic handgrip exercise. Furthermore, enhanced arterial vasodilation (FBF and FVC) was correlated with improvements in FMD and muscle oxygenation. Our findings indicate that CIT supplementation improves muscle oxygenation and oxygen extraction responses to exercise by increasing local artery endothelial-dependent vasodilation in hypertensive postmenopausal women.

Aging and hypertension cause endothelial dysfunction due to the structural and functional changes in limb arteries and arterioles [10]. Specifically, brachial artery endothelial function progressively declines in women across the menopause transition due to ARG deficiency [8]. Reduced NO production and increased vasoconstrictors (e.g., catecholamines and endothelin) in postmenopausal women contribute to the development of hypertension [38]. Postmenopausal women have a higher prevalence of systolic hypertension than men due to the proximal aortic stiffness [39,40], leading to greater risk of heart failure [40]. In this study, we observed that CIT supplementation reduced resting brachial and aortic SBP by 4 mmHg and 3 mmHg, respectively. Moreover, although there was no change in aortic stiffness, we found that 4 weeks of CIT supplementation improved FMD by 1.6%, showing the clinical significance of a potential reduction in the risk of cardiovascular disease by approximately 19% [41]. Similarly, previous studies have reported the efficacy of CIT supplementation on endothelial function in postmenopausal women [30,42] but not on aortic stiffness [43,44]. Our findings suggest that oral CIT supplementation may lead to functional improvement in limb arteries by enhancing the ARG-NO pathway [30,31], despite no change in the structural property of the conduit arteries.

Muscle BF is regulated by a balance between vasodilation and vasoconstriction [45]. During exercise, contracting muscles require greater BF and oxygen delivery to capillaries in order to meet an elevated metabolic demand [15,46], which primarily occurs via local vasodilation in limb arteries and arterioles [12,47]. However, arterial BF during exercise is blunted in older adults [34,48] due, in part, to reduced NO-mediated endothelial vasodilation [34,49,50]. In the current study, we found that 4 weeks of CIT (10 g/day) supplementation increased FBF and FVC during handgrip exercise by 47% and 49%, respectively, in hypertensive postmenopausal women. These findings are in line with a previous study showing that acute dietary nitrate, an NO donor, enhanced FBF and FVC during handgrip exercise in young healthy adults [36]. On the other hand, CIT supplementation (6 g/day) for 7 days failed to increase FVC during handgrip exercise performed at 10% of MVC in young, healthy women [51], suggesting that CIT does not improve arterial vasodilation at a low workload in women with apparently normal endothelial function [13]. In addition, 2 weeks of CIT (6 g/day) supplementation increased leg BF and VC during calf exercise in older men, but not in apparently healthy older women [52]. Despite the use of CIT, the disparity between the previous [52] and present findings in older women may be attributed to the short duration of the intervention (2 and 4 weeks), lower CIT dose (6 and 10 g), and non-hypertensive status (normotensives and hypertensives), and lower NO-dependent vasodilator response in leg compared to arm arteries [53].

Aging and hypertension augment muscle sympathetic nerve activity and local vasoconstriction during exercise, reducing BF to active skeletal muscles [48,50]. SBP responses to exercise are augmented by menopause and hypertension [54,55]. A greater increase in SBP during exercise in older women, compared to young women and men, and older men is due to an inability to attenuate vasoconstriction [55]. Postmenopausal women have exaggerated SBP responses to exercise due, in part, to endothelial dysfunction and increased sympathetic-mediated vasoconstriction [33,56,57]. Although several endothelial-derived and skeletal muscle-derived (ATP, adenosine, potassium) factors are involved in augmenting local BF, NO and prostaglandin are the main vasodilators during exercise. In humans, the infusion of inhibitors of NO and prostaglandin synthesis resulted in reduced BF during exercise [58,59]. However, NO caused a consistent contribution to forearm hyperemia while prostaglandin had a modest and transient vasodilator effect [58]. Moreover, the inhibition of endothelial-derived hyperpolarizing factors did not affect exercise BF [59]. Therefore, the imbalance between local vasodilation and sympathetic vasoconstriction during exercise can be explained, in part, by impaired NO availability [15]. Improved leg BF and VC responses to exercise after CIT supplementation in older men may be influenced by a reduced MAP during exercise due to attenuated sympathetic vasomotor activity [52]. In the present study, CIT supplementation increased FBF and FVC during exercise without a significant change in MAP; thus, improved ARG and NO bioavailability to produce vascular smooth muscle relaxation may be the most likely mechanism. Our group recently demonstrated that increases in leg BF and VC during exercise were strongly associated with endothelial-mediated vasodilation in non-obese postmenopausal women [34]. In contrast, obesity attenuated BF and VC responses to exercise. Moreover, we previously reported that CIT supplementation improved brachial artery endothelial function and ARG availability in hypertensive postmenopausal women [30]. Taken together, our findings suggest that CIT supplementation can enhance arm artery vasodilation during low-intensity exercise in hypertensive postmenopausal women, which may be due to improved endothelial function [30,34] and related sympathetic-mediated vasoconstriction attenuation [48] commonly known as functional sympatholysis.

Microvascular function is important for the delivery of oxygen and nutrients to skeletal muscles [60,61]. In individuals with hypertension, structural and functional microvascular abnormalities contribute to reduced oxygen delivery during exercise due to increased sympathetic-mediated vasoconstriction and reduced vasodilatory capacity [62,63]. In the present study, CIT supplementation improved forearm muscle TSI, O_2_Hb, and HHb levels during low-intensity handgrip exercise. Consistent with our findings, 7 days of CIT supplementation (6 g daily) raised TSI and attenuated the increase in HHb during moderate-intensity cycling exercise in young healthy males, suggesting improved oxygen availability within the muscle microvasculature by increasing muscle perfusion and reducing oxygen extraction [25]. Similarly, an improvement in vastus lateralis TSI during moderate-intensity cycling exercise was observed after 2 weeks of watermelon juice, a naturally rich source of CIT, in active young men [64]. These improvements may be explained by increased oxygen delivery coupled with reduced oxygen extraction during exercise [18,65,66], suggesting improved oxygen efficiency and oxygen cost [18,67]. At the same relative intensity, a greater BP response is required for achieving similar muscle TSI during exercise in hypertensive compared to normotensive adults due to less capillary density, attenuated vasodilation, and exaggerated sympathetic-mediated vasoconstriction [35]. In the present study, there was a significant improvement in muscle oxygenation during exercise without an excessive BP response in postmenopausal women with hypertension after CIT supplementation compared to placebo. This could be explained by greater local artery vasodilation providing improved perfusion to contracting skeletal muscles [14], thereby increased oxygen delivery [25]. Taken together, 4 weeks of CIT supplementation was effective to improve muscle oxygenation and extraction during exercise in hypertensive postmenopausal women. Improved microvascular function and muscle oxygenation may enhance exercise capacity and performance [18] in hypertensive postmenopausal women. This is the first study to demonstrate the effects of a longer and higher dose of CIT supplementation on muscle oxygenation during low-intensity exercise in hypertensive postmenopausal women, whereas most of the previous studies examined shorter supplementation in young healthy men [25,64]. Endothelial dysfunction and hypertension are independent precursors to the increased risk of cardiovascular disease in postmenopausal women [68,69]. Our findings are clinically important considering that 4 weeks of CIT improved resting BP, endothelial-mediated vasodilation, and vasodilatory capacity during exercise, suggesting a reduction in future cardiovascular risk in this vulnerable population.

There are some limitations in the present study. First, we did not report ARG and NO bioavailability in this study. However, the present data were part of a previous study from our group that demonstrated improved endothelial function via enhanced ARG bioavailability after CIT supplementation in hypertensive postmenopausal women [30]. Further, we used NIRS as a non-invasive method to evaluate augmented skeletal muscle oxygenation during dynamic exercise [66], which may have been influenced by NO and muscle metabolites (e.g., ATP, adenosine) [70]. Accordingly, the improvements in arterial vasodilation and muscle oxygenation during exercise found after CIT may be associated with improved macro-and microvascular endothelial function at rest [30,31]. Second, we did not measure the subcutaneous adipose tissue thickness of the forearm using skinfolds, which may reduce absolute NIRS signals [71]. However, the forearm (around flexor digitorum profundus) has a relatively lower subcutaneous fat than other sites [72]. In addition, since NIRS-derived measures in forearm muscles were not affected in obese individuals with higher BMI (32.9 ± 1.9 kg/m^2^) than our participants [73], the influence of adipose tissue thickness on NIRS signal in our study can be neglected. Third, we did not measure serum Hb levels, which may affect NIRS-derived measures. Lastly, this study was conducted in hypertensive postmenopausal women and the present findings cannot be extrapolated to other populations. Future studies are needed to investigate the chronic longer effects of CIT supplementation on arm and leg macro- and microvascular function at rest and during exercise in various clinical populations with cardiometabolic risk factors or diseases.

## 5. Conclusions

Four weeks of CIT supplementation increased brachial artery endothelial function and vasodilatory capacity during low-intensity handgrip exercise in hypertensive postmenopausal women. Our findings suggest that CIT supplementation can enhance peripheral artery vasodilation, leading to improvements in skeletal muscle oxygenation during exercise.

## Figures and Tables

**Table 1:** Participant characteristics and medications.

Characteristics	Placebo (*n* = 11)	CIT (*n* = 11)	*p*
Age (years)	64 ± 5	61 ± 7	0.35
Height (m)	1.58 ± 0.09	1.59 ± 0.05	0.45
Weight (kg)	74.4 ± 15.7	75.0 ± 10.8	0.92
Body mass index (kg/m^2^)	30.2 ± 5.9	29.8 ± 4.0	0.85
Waist circumference (cm)	97.5 ± 18.3	90.8 ± 10.4	0.30
MVC (kg)	30 ± 7	30 ± 6	0.92
*Medication (n)*			
ARB	1	2	
ACE inhibitors	0	1	
Diuretics	1	1	
Calcium channel blockers	1	0	
Statin	1	1	

Values are the mean ± SD or number (*n*). Abbreviations: CIT, L-citrulline; MVC, maximal voluntary contraction; ARB, angiotensin receptor blocker; ACE, angiotensin converting enzyme. *p*-values are the between-group differences from the *t*-test.

**Table 2:** Vascular function and blood pressure at rest, and blood pressure responses to exercise at 0 and 4 weeks.

Variables	Placebo (*n* = 11)			CIT (*n* = 11)			*p*
	0 Week	4 Weeks	Δ0 to 4 Weeks	0 Week	4 Weeks	Δ0 to 4 Weeks	
*Rest*							
cfPWV (m/s)	9.2 ± 1.3	8.6 ± 1.6	Δ−0.6 ± 0.8	9.4 ± 2.0	8.5 ± 1.1 *	Δ−0.9 ± 1.3	0.46
Baseline Diameter (mm)	3.67 ± 0.48	3.81 ± 0.51	Δ0.14 ± 0.21	3.64 ± 0.43	3.60 ± 0.32	Δ−0.04 ± 0.34	0.14
Peak Diameter (mm)	3.82 ± 0.32	3.98 ± 0.53	Δ0.13 ± 0.18	3.83 ± 0.44	3.82 ± 0.32	Δ−0.01 ± 0.38	0.31
Brachial FMD (%)	4.84 ± 1.75	4.18 ± 2.19	Δ−0.7 ± 2.1	5.03 ± 2.34	6.62 ± 2.22 *^†^	Δ1.6 ± 2.2	0.02
Baseline Shear Rate (s^−1^)	125 ± 45	131 ± 36	Δ6 ± 32	125 ± 46	169 ± 61	Δ43 ± 164	0.47
Peak Shear Rate (s^−1^)	1028 ± 264	1016 ± 302	Δ−12 ± 209	1055 ± 245	1148 ± 401	Δ92 ± 428	0.78
FMD/Shear Rate_AUC_ (u.a.)	2.18 ± 1.23	1.82 ± 1.29	Δ−0.36 ± 1.55	1.52 ± 0.83	2.40 ± 1.06	Δ0.88 ± 1.35	0.06
Brachial SBP (mmHg)	133 ± 14	135 ± 15	Δ2 ± 5	132 ± 12	128 ± 8 *^†^	Δ−4 ± 6	0.04
Brachial DBP (mmHg)	78 ± 10	79 ± 11	Δ2 ± 3	82 ± 8	81 ± 7	Δ−1 ± 4	0.09
Brachial MAP (mmHg)	96 ± 10	98 ± 11	Δ2 ± 4	99 ± 8	97 ± 6	Δ−2 ± 4	0.04
Brachial PP (mmHg)	55 ± 13	55 ± 12	Δ1 ± 5	50 ± 9	47 ± 8	Δ−2 ± 7	0.21
Aortic SBP (mmHg)	127 ± 13	130 ± 15	Δ3 ± 5	126 ± 10	123 ± 6 *^†^	Δ−4 ± 6	0.01
Aortic DBP (mmHg)	78 ± 11	79 ± 12	Δ1 ± 4	83 ± 8	80 ± 7 *	Δ−3 ± 6	0.06
Aortic MAP (mmHg)	95 ± 10	96 ± 12	Δ1 ± 4	95 ± 8	94 ± 6 *^†^	Δ−3 ± 5	0.02
Aortic PP (mmHg)	49 ± 12	51 ± 11	Δ2 ± 3	43 ± 7	43 ± 7	Δ0 ± 6	0.28
*Exercise*							
Δ Brachial SBP (mmHg)	19 ± 10	19 ± 10	Δ0 ± 10	20 ± 9	22 ± 7	Δ2 ± 8	0.71
Δ Brachial DBP (mmHg)	6 ± 10	8 ± 5	Δ2 ± 9	8 ± 7	9 ± 8	Δ1 ± 6	0.73
Δ Brachial MAP (mmHg)	10 ± 10	11 ± 6	Δ1 ± 8	12 ± 6	13 ± 7	Δ1 ± 4	0.96
Δ Brachial PP (mmHg)	13 ± 8	12 ± 7	Δ−2 ± 11	12 ± 10	13 ± 7	Δ1 ± 11	0.58

Values are the mean ± SD. Abbreviations: CIT, L-citrulline; cfPWV, carotid–femoral pulse wave velocity; FMD, flow-mediated dilation; AUC, area under the curve; SBP, systolic blood pressure; DBP, diastolic blood pressure; MAP, mean arterial pressure; PP, pulse pressure; Δ, change from rest to the last minute of exercise. *p*-values are the group-by-time interaction from two-way repeated measures ANOVA. * *p* < 0.05 vs. 0 week; ^†^ *p* < 0.05 vs. placebo.

**Table 3:** Brachial artery vasodilation capacity at rest and responses to handgrip exercise at 0 and 4 weeks.

Variables	Placebo (*n* = 11)				CIT (*n* = 11)			
	0 Week		4 Weeks		0 Week		4 Weeks	
*FBF* (mL/min)								
Rest	68 ± 41	-	107 ± 105	-	62 ± 18	-	90 ± 90	-
1 min	150 ± 47 *	Δ82 ± 43 *	172 ± 115 *	Δ65 ± 39 *	127 ± 40 *	Δ65 ± 30 *	189 ± 127 *	Δ100 ± 52 *^†^
2 min	176 ± 41 *	Δ107 ± 35 *	204 ± 125 *	Δ98 ± 35 *	149 ± 54 *	Δ87 ± 46 *	225 ± 133 *	Δ135 ± 65 *^†^
3 min	200 ± 35 *	Δ132 ± 21 *	212 ± 112 *	Δ106 ± 22 *	177 ± 51 *	Δ115 ± 38 *	265 ± 136 *	Δ176 ± 77 *^†^
Average over 3 min	175 ± 38 *	Δ107 ± 30 *	191 ± 116 *	Δ90 ± 26 *	151 ± 44 *	Δ89 ± 33 *	226 ± 126 *	Δ137 ± 53 *^†^
*FVC* (mL/min/mmHg)								
Rest	68 ± 44		64 ± 19		110 ±116		91 ± 82	
1 min	133 ± 44 *	Δ65 ± 41 *	120 ± 43 *	Δ46 ± 36	156 ± 116 *	Δ56 ± 31 *	177 ± 112 *	Δ86 ± 43 *^†^
2 min	155 ± 39 *	Δ87 ± 33 *	140 ± 53 *	Δ74 ± 19 *	184 ± 118 *	Δ76 ± 43 *	206 ± 117 *	Δ115 ± 54 *^†^
3 min	176 ± 37 *	Δ108 ± 23 *	161 ± 53 *	Δ79 ± 16 *	189 ± 113 *	Δ97 ± 40 *	240 ± 125 *	Δ149 ± 69 *^†^
Average over 3 min	155 ± 38 *	Δ87 ± 30 *	176 ± 115 *	Δ92 ± 42 *	140 ± 47 *	Δ76 ± 35 *	208 ± 113 *	Δ117 ± 44 *^†^

Values are the mean ± SD. Abbreviations: CIT, L-citrulline; FBF, forearm blood flow; FVC, forearm vascular conductance; Δ, change from rest to exercise. * *p* < 0.05 vs. rest; ^†^ *p* < 0.05 vs. placebo the same week.

**Table 4:** Forearm muscle oxygenation during handgrip exercise at 0 and 4 weeks.

Variables	Placebo (*n* = 11)				CIT (*n* = 11)			
	0 Week		4 Weeks		0 Week		4 Weeks	
*TSI* (%)								
Rest	61.4 ± 2.3	-	59.6 ± 2.7	-	61.8 ± 4.1	-	60.6 ± 3.3	-
1 min	61.5 ± 2.5	Δ0.18 ± 1.24	58.8 ± 3.8	Δ−0.86 ± 2.27	61.2 ± 5.6	Δ−0.65 ± 2.12	61.6 ± 3.2	Δ0.98 ± 1.21 ^†^
2 min	60.6 ± 2.2	Δ−0.71 ± 1.46	58.0 ± 4.2	Δ−1.64 ± 2.75	60.2 ± 5.7	Δ−1.60 ± 2.66	60.6 ± 3.2	Δ0.01 ± 1.96 ^†^
3 min	60.5 ± 4.2	Δ−0.82 ± 1.37	58.3 ± 4.1	Δ−1.36 ± 2.39	60.4 ± 5.6	Δ−1.46 ± 2.51	60.7 ± 3.2	Δ0.13 ± 1.73 ^†^
Average over 3 min	60.9 ± 2.2	Δ−0.45 ± 1.07	58.4 ± 4.0	Δ−1.29 ± 2.42	60.6 ± 5.6	Δ−1.24 ± 2.38	61.0 ± 3.2	Δ0.37 ± 1.62 ^†^
*HHb* (μM)								
Rest	−0.60 ±0.62	-	−0.95 ± 0.94	-	−0.38 ± 0.49	-	−0.51 ± 0.56	-
1 min	0.96 ± 1.48	Δ1.56 ± 1.70	1.48 ± 2.99 *	Δ2.43 ± 2.63 *	2.01 ± 2.07 *	Δ2.39 ± 2.10 *	−0.69 ± 1.96	Δ−0.18 ± 1.57 ^†^
2 min	1.64 ± 1.45 *	Δ2.24 ± 1.63 *	2.80 ± 3.73 *	Δ3.76 ± 3.68 *	2.97 ± 2.92 *	Δ3.35 ± 2.97 *	−0.01 ± 2.48 ^†^	Δ0.49 ± 2.13 ^†^
3 min	2.45 ± 2.82 *	Δ3.04 ± 2.54 *	2.80 ± 3.98 *	Δ3.75 ± 3.90 *	2.93 ± 3.10 *	Δ3.30 ± 3.19 *	−0.06 ± 2.23 ^†^	Δ0.45 ± 1.87 ^†^
Average over 3 min	1.68 ± 1.57 *	Δ2.28 ± 1.66 *	2.36 ± 3.43 *	Δ3.31 ± 3.27 *	2.64 ± 2.65 *	Δ3.02 ± 2.71 *	−0.25 ± 2.20 ^†^	Δ0.25 ± 1.83 ^†^
*O_2_Hb* (μM)								
Rest	0.23 ± 0.63	-	0.35 ± 0.76	-	0.45 ± 0.47	-	0.70 ± 0.47	-
1 min	1.08 ± 3.11	Δ0.85 ± 3.07	0.17 ± 2.60	Δ−0.18 ± 2.30	1.43 ± 4.06	Δ0.97 ± 4.00	3.22 ± 2.42 *^†^	Δ2.52 ± 2.34 *^†^
2 min	1.06 ± 3.34	Δ0.83 ± 3.44	−0.85 ± 3.99	Δ−1.20 ± 3.81	0.21 ± 5.77	Δ−0.24 ± 5.73	3.45 ± 3.15 ^†^	Δ2.75 ± 3.11 ^†^
3 min	1.55 ± 3.77	Δ2.32 ± 3.79	−0.32 ± 3.75	Δ−0.67 ± 3.51	0.62 ± 5.91	Δ0.17 ± 5.93	3.71 ± 2.88 *^†^	Δ3.01 ± 2.86 *^†^
Average over 3 min	1.56 ± 3.33	Δ1.34 ± 3.36	−0.34 ± 3.32	Δ−0.69 ± 3.08	0.75 ± 5.17	Δ0.30 ± 5.14	3.26 ± 2.79 *^†^	Δ2.76 ± 2.75 *^†^

Values are the mean ± SD. Abbreviations: CIT, L-citrulline; TSI, tissue oxygen saturation index; HHb, deoxygenated hemoglobin; O_2_Hb, oxygenated hemoglobin; Δ, change from rest to exercise. * *p* < 0.05 vs. rest; ^†^ *p* < 0.05 vs. placebo the same week.

## Data Availability

The original contributions presented in the study are included in the article, further inquiries can be directed to the corresponding author.
